# Aberrant brain intra- and internetwork functional connectivity in children with Prader-Willi syndrome

**DOI:** 10.1007/s00234-023-03259-x

**Published:** 2023-11-25

**Authors:** Zhongxin Huang, Xiangmin Zhang, Xinyi Yang, Shuang Ding, Jinhua Cai

**Affiliations:** 1 Department of Radiology, Children’s Hospital of Chongqing Medical University, No. 136, Zhongshan Second Road 400014, Chongqing, China; 2 Ministry of Education Key Laboratory of Child Development and Disorders, Chongqing, 400014 China; 3 Chongqing Key Laboratory of Translational Medical Research in Cognitive Development and Learning and Memory Disorders, Chongqing, 400014 China

**Keywords:** Prader-Willi syndrome, Magnetic resonance imaging, Functional connectivity, Independent component analysis

## Abstract

**Purpose:**

Prader-Willi syndrome (PWS) suffers from brain functional reorganization and developmental delays during childhood, but the underlying neurodevelopmental mechanism is unclear. This paper aims to investigate the intra- and internetwork functional connectivity (FC) changes, and their relationships with developmental delays in PWS children.

**Methods:**

Resting-state functional magnetic resonance imaging datasets of PWS children and healthy controls (HCs) were acquired. Independent component analysis was used to acquire core resting-state networks (RSNs). The intra- and internetwork FC patterns were then investigated.

**Results:**

In terms of intranetwork FC, children with PWS had lower FC in the dorsal attention network, the auditory network, the medial visual network (VN) and the sensorimotor network (SMN) than HCs (FWE-corrected, *p* < 0.05). In terms of internetwork FC, PWS children had decreased FC between the following pairs of regions: posterior default mode network (DMN) and anterior DMN; posterior DMN and SMN; SMN and posterior VN and salience network and medial VN (FDR-corrected, *p* < 0.05). Partial correlation analyses revealed that the intranetwork FC patterns were positively correlated with developmental quotients in PWS children, while the internetwork FC patterns were completely opposite (*p* < 0.05). Intranetwork FC patterns showed an area under the receiver operating characteristic curve of 0.947, with a sensitivity of 96.15% and a specificity of 81.25% for differentiating between PWS and HCs.

**Conclusion:**

Impaired intra- and internetwork FC patterns in PWS children are associated with developmental delays, which may result from neural pathway dysfunctions. Intranetwork FC reorganization patterns can discriminate PWS children from HCs.

**Registration number on the Chinese Clinical Trail Registry:**

ChiCTR2100046551.

## Introduction

Prader-Willi syndrome (PWS) is the first known epigenetic disorder (OMIM #176270) and involves the imprinted chromosomal domain of the parental 15q11.2-q13.3 allele [1]. The prevalence of PWS is approximately 1/30,000 per year in Europe [2]. PWS has a wide range of clinical manifestations, which mainly related to cognitive, endocrine, and behavioral abnormalities. Individuals with PWS experience relatively predictable nutritional phases, starting with poor feeding in infancy and progressing to excessive appetite in school-age children, having profound effects on cognitive, behavioral, and intellectual development [3]. However, the underlying neurodevelopmental mechanisms of cognitive and behavioral abnormalities in PWS remain unknown.

Various imaging techniques, such as electroencephalography, positron emission computed tomography, and magnetic resonance imaging (MRI), have been used to assess neural impairment in the brains of PWS patients [4]. Resting-state functional MRI (rs-fMRI) is a noninvasive tool for measuring coactivated blood oxygen level-dependent signals to assess neuronal activity. Functional connectivity (FC) mainly refers to the indirect analysis of spontaneous brain activity signals, including seed-point based FC, independent component analysis (ICA) and complex brain network analyses. Recently, rs-fMRI related research has focused on central nervous system theories to explain obesity and obsessive–compulsive behavior in PWS [5]. Holsen et al. [6] enrolled 9 patients with PWS and found hyperfunction after eating in limbic/paralimbic regions (e.g., the amygdala) and the suppress food intake regions (e.g., the medial prefrontal cortex) by fMRI method. Pujol et al. [7] then selected basal ganglia as seeds to calculated whole brain FC maps, illustrating that the obsessive–compulsive behavior about PWS was associated with alterations in prefrontal loop. However, most prior studies focused on local abnormal brain activity or FC and not considered changes in large-scale network FC in PWS.

ICA is a data-driven multivariate blind source separation approach. The advantages of ICA over other methods for constructing functional brain networks (e.g., a priori seed points) are obvious. ICA does not require any prior assumptions to intuitively explore the temporal correlations of resting-state MRI data. The majority of the components extracted by ICA represent specific cognitive functional networks. ICA separates rs-fMRI data into spatially independent patterns of brain activity [8], called resting-state networks (RSNs). These RSNs are used for subsequent intra- and internetwork FC analyses. Intranetwork FC of core RSNs is extracted based on the temporal correlations among brain regions, and internetwork FC is extracted based on the temporal correlations across different RSNs. In recent years, intranetwork and internetwork analyses have been used quickly and effectively to study the pathogenesis of a variety of developmental disorders and psychiatric illnesses, such as autism spectrum disorders, epilepsy and depressive disorder [9–12]. However, ICA studies in PWS children to examine abnormal changes in intra- and inter-RSNs are uncommon.

In the current study, we examined how altered network connectivity patterns affect developmental performance and explored possible neuroimaging-based biomarkers to guide personalized diagnosis of PWS. First, we used ICA-based RSN analysis to identify altered intranetwork/internetwork FC in PWS children and their relationships with developmental performance. Then, we also generated receiver operating characteristic (ROC) curves to explore whether altered intranetwork FC can be used as sensitive and specific biomarkers for PWS screening and prognosis.

## Materials and Methods

The protocol for this study was approved by the Human Research Ethics Committee of our hospital (2021 Grant No. 162). All study procedures followed the Declaration of Helsinki [13]. Prior to inclusion, written informed consent was acquired from parents of each participant. The registration number on the Chinese Clinical Trail Registry is ChiCTR2100046551.

### Participants

We recruited children with PWS from the Endocrinology and Rehabilitation Departments of our hospital from May 2021 to October 2022. Enrolled PWS patients were selected based on the following criteria: (a) genetically confirmed PWS [14] and (b) aged 2 to 6 years. The following were the exclusion criteria: (a) neurological or psychiatric history, (b) history of psychotropic medication, and (c) MRI contraindications. All PWS patients underwent evaluation with the Griffiths Development Scales (GDS). We also advertised to recruite a group of age- and sex-matched healthy controls (HCs). Through multiple assessments by pediatricians, all recuited HCs were considered typically developmental children with reports of normal growth and development, normal intelligence, and normal neurological examinations. The exclusion criteria of the HCs are the same as those of the PWS group. Following MRI scans and data preprocessing, three children with PWS and four HCs were excluded due to excessive head movements (translation > 2.0 mm or rotation > 2° [15]). Ultimately, this study included 58 subjects: 26 children with PWS (17 males; mean age ± SD: 42.04 ± 22.23 months) and 32 HCs (21 males; mean age ± SD: 44.88 ± 16.52 months).

### MRI data acquisition

All MRI data were acquired using a 3.0 Tesla MR scanner (Philips Achieva) with an 8-channel head coil. To limit head motion and improve the quality of neuroimages, PWS group and HCs were under moderate sleep deprivation and achieved with intravenous administration of propofol (loading dose of 1 mg/kg, followed by 200–300 μg/kg/min). During data acquisition, foam pads were used to reduce head motion, and earplugs were used to reduce scanner noise.

T1-weighted anatomical imaging was acquired using a three-dimensional turbo field echo (TFE) sequence. The parameters were as follows: repetition time (TR) = 7.4 ms, echo time (TE) = 3.8 ms, slice thickness = 1 mm, gap = 0 mm, slices = 260, field of view (FOV) = 250 mm × 250 mm, acquisition matrix = 228 × 227, flip angle = 8°, voxel size = 0.60 mm × 1.04 mm × 1.04 mm, and total time = 4 min 16 s. The rs-fMRI sequence was acquired using a echo planner imaging (EPI) sequence. The parameters were as follows: TR = 2000 ms, TE = 35 ms, slice thickness = 4 mm, slices = 33, FOV = 240 mm × 240 mm, acquisition matrix = 80 × 78, flip angle = 90°, voxel size = 3.75 mm × 3.75 mm × 4 mm with no gap, and total time = 8 min 06 s.

### MRI data preprocessing

For preprocessing MRI data, we used the batch-processing tool Data Processing & Analysis for Brain Imaging (DPABI; http://www.rfmri.org/dpabi) [16] based on Statistical Parametric Mapping 12 (SPM12; http://www.fifil.ion.ucl.ac.uk/spm/software/spm12/) software in MATLAB 2013b (MathWorks Inc., Natick, MA, USA). The following were the main steps: (a) the first ten volumes from each functional time series were discarded; (b) slice-timing correction and realignment for head motion correction were performed; (c) the corrected data set was normalized to the Montreal Neurological Institute (MNI) template and resampled to a resolution of 3-mm cubic voxel; (d) the covariates were regressed from the time course of each voxel, including head motion parameters in 24 directions and four average confounding signals (cerebrospinal fluid, white matter, gray matter and the whole brain); and (e) the full width at half maximum of 6 mm of the Gaussian kernel was used to smooth the data set.

### Independent component analysis

The preprocessed MRI data set was then parcellated by ICA using the GIFT toolbox (Version 4.0b, http://mialab.mrn.org/software/gift/). This procedure comprised following steps: (a) the number of independent components (N = 36) was automatically estimated based on the minimum description length criteria; (b) the data set was decomposed into linear mixtures of spatially independent components with unique time course profiles using spatial ICA; (c) to reduce the dimensionality, after concatenating data from all the participants into a single data set, principal component analysis was performed, in which the infomax algorithm was iterated 100 times using ICASSO (http://research.ics.tkk.fi/ica/icasso/) to ensure the stability and consistency [17, 18]; (d) the group ICA back-reconstruction approach was used to compile spatial maps and time courses for individual subjects; and (e) the spatial maps were converted into a z score after the reverse reconstruction [19].

Subsets of the independent components were identified through the spatial correlation with previously published corresponding template [20, 21]. In total, 12 functional networks were selected from the 36 independent components as our RSNs for subsequent intra- and internetwork FC analysis.

### Data statistical analysis

Demographic data for all subjects and GDS scale quotients for those with PWS were obtained. First, after determining that the data were normally distributed, we evaluated differences in the demographic data of children with PWS and HCs groups using two-sample *t* tests and chi-square tests (SPSS 24.0; IBM, Armonk, NY, USA). *P* < 0.05 indicated significance. Then, all the PWS subjects underwent the GDS assessment [22, 23]. In the GDS assessment, the General Quotient (GQ) measures intelligence, while the other six subscales measure motor, language, eye-hand coordination, social, performance and reasoning abilities. When the subscale quotients or GQ were at least two standard deviations below the mean (subscale quotients or GQ < 70), developmental delays were indicated [24].

We created a sample-specific spatial map for each component. A one-sample *t* test with a familywise error (FWE)-corrected (*p* < 0.05) and with a cluster size > 100 voxels [25] was performed on each independent component of PWS and HC based on SPM12. For each component, we obtained a total mask by further combining the masks for each PWS and HC. A two-sample *t* test (FWE-corrected *p* < 0.05 and a cluster size > 10 voxels) with regressing covariates (age and sex) was used to correct the comparison results [25].

By computing the temporal correlation across different RSNs, we investigated fluctuations in the patterns of internetwork FC. First, we used ICA to obtain the time course of each RSN. We then computed the temporal correlation, the functional between-network connectivity, using the time courses of each pair of the 12 RSNs. The resulting functional between-network connectivity were then normalized using the Fisher r-to-z transformation to meet a normal distribution and obtain a 12 × 12 matrix internetwork FC within RSNs. Finally, we used a two-sample *t* test to compare FC differences between the two groups, the false discovery rate (FDR) method (*p* < 0.05) and regressing covariates (age and sex) was used to correct the comparison results [26].

Correlation analyses were conducted to determine and quantify the relationships between intra- and internetwork FC and developmental scales using SPSS software. After controlling for certain variables (e.g., age and sex), we performed a two-tailed partial correlation analysis; *p* < 0.05 indicated statistical significance.

We extracted the intranetwork FC of each core region from the RSNs of each participant. Then, ROC curves were constructed to examine sensitivity, specificity, and the area under the curve (AUC) for intranetwork FC of these core regions. Combining all of the above core regions, we again calculated these parameters using the same method. Finally, AUC from single and combined core regions calculated from intranetwork FC was compared in distinguishing PWS patients from the HC group. These analyses were carried out using GraphPad Prism 9.0 software (GraphPad Prism Software, San Diego, USA).

## Results

### Demographic characteristics and developmental features

The demographic characteristics of the participants (PWS and HCs) are shown in Table 1. No significant between-group differences were observed in age, sex, height or weight (*p* > 0.05). The percentages of children exhibiting delays in different domains of the GDS are also shown in Table 1.

**Table 1:** Demographic and clinical characterstics of participants

Variables	PWS (*n* = 26)		HCs (*n* = 32)
Age (months)	42.04 ± 22.23		44.88 ± 16.52
Male *n* (%)	17 (65.38%)		21 (65.63%)
Height (cm)	92.63 ± 13.74		96.47 ± 12.33
Weight (kg)	17.49 ± 8.15		15.73 ± 4.07
GDS Quotient (subscale letter label)		Delay^a^ *n* (%)	
General (GQ)	61.27 ± 13.50	19 (73.08%)	
Locomotor (AQ)	59.26 ± 20.02	19 (73.08%)	
Personal-social (BQ)	67.30 ± 21.70	14 (53.85%)	
Hearing and language (CQ)	63.80 ± 16.50	17 (65.38%)	
Eye-hand corordination (DQ)	69.60 ± 23.80	14 (53.85%)	
Performance (EQ)	64.58 ± 19.55	16 (61.54%)	
Practical reasoning (FQ)^b^	80.93 ± 26.16	1/8 (12.50%)	

All data given as mean ± standard deviation or n (%)

^a^A GQ or a subscale quotient < 70

^b^FQ was measured for n children (2–6 year olds) in the sample

*p* value for two-way unpaired* t*-test or chi-squared test

^**^ for* p* < 0.01

BP, binding proteins; GDS, Griffith Development Scales; HCs, healthy controls; PWS, Prader-Willi syndrome

### Meaningful components

**Fig. 1:**
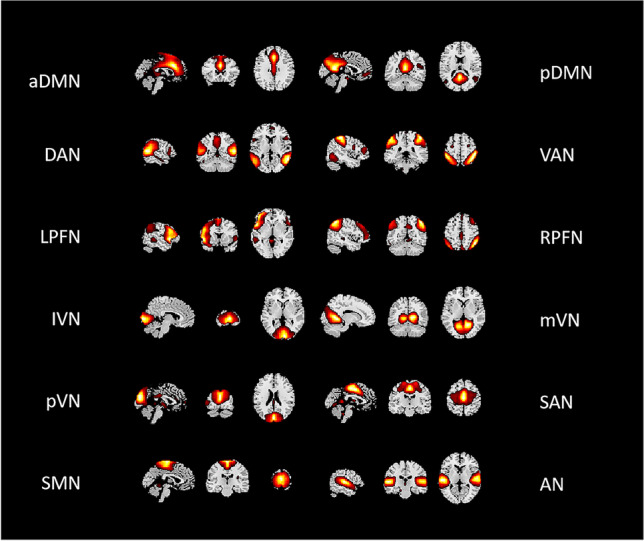
The 12 core RSNs selected by the independent component analysis. aDMN, anterior default mode network; AN, auditory network; DAN, dorsal attention network; lFPN, left frontoparietal network; lVN, lateral visual network; mVN, medial visual network; pDMN, posterior default mode network; pVN, posterior visual network; rFPN, right frontoparietal network; RSNs, resting-state networks; SAN, salience network; SMN, sensorimotor network; VAN, ventral attention network

Of the 36 independent components estimated by the GIFT toolbox, 12 were identified as RSNs in the current study (Fig. 1). These RSNs included the anterior and posterior default mode networks (aDMN and pDMN); dorsal and ventral attention networks (DAN and VAN); left and right frontoparietal networks (lFPN and rFPN); medial, lateral, and posterior visual networks (mVN, lVN, and pVN); salience network (SAN); sensorimotor network (SMN) and auditory network (AN).

### Altered intranetwork functional connectivity

**Fig. 2:**
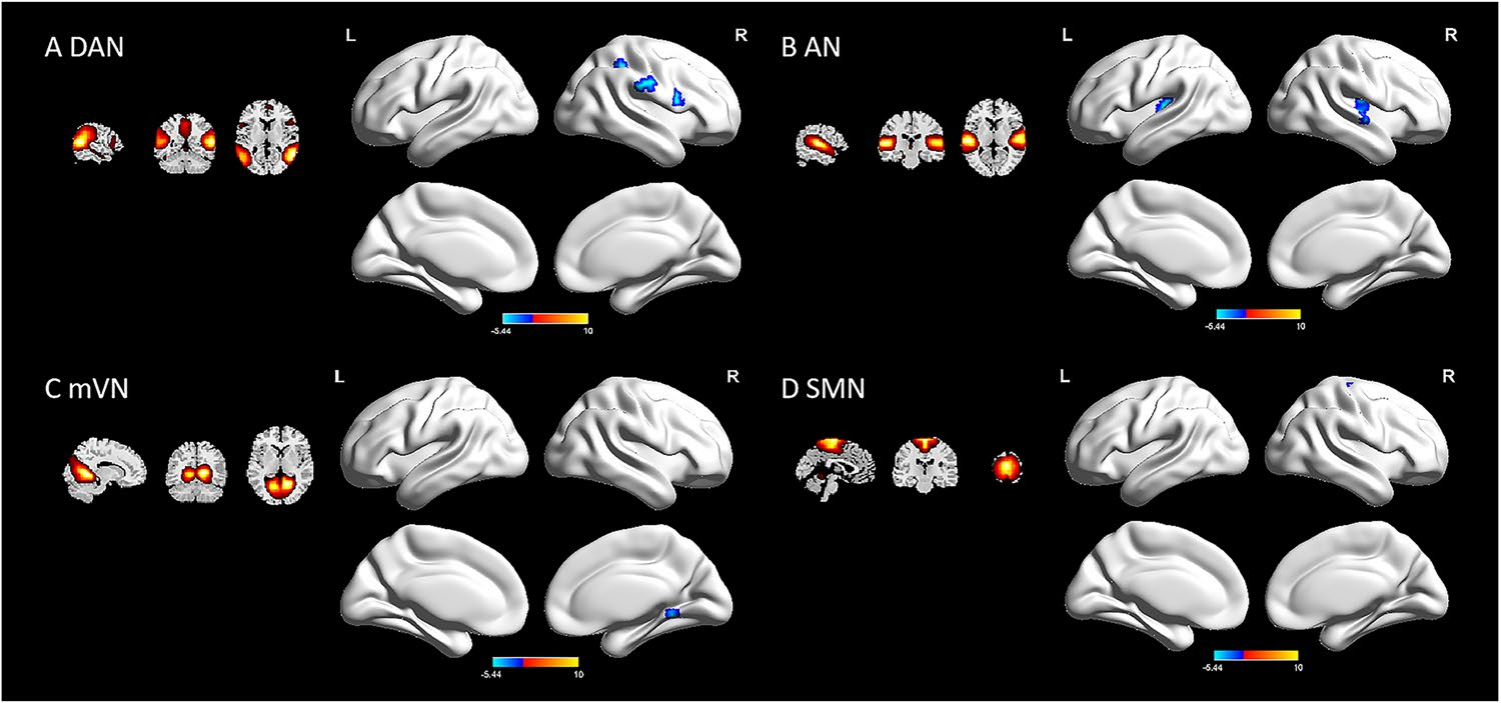
PWS group showed a decrease in intranetwork functional connectivity compared to HCs by two-sample *t*-test (FWE-corrected *p* < 0.05 and a cluster size > 10 voxels). **A** Decreased core regions in DAN. **B** Decreased core regions in AN. **C** Decreased core region in mVN; and **D** Decreased core region in SMN. AN, auditory network; DAN, dorsal attention network; FWE, the familywise error; HCs, healthy controls; L, left; mVN, medial visual network; PWS, Prader-Willi syndrome; R, right; SMN, sensorimotor network

In the PWS group, four RSNs displayed significant decreased intranetwork FC differences compared with those of HCs. The following were decreased core regions of the four RSNs: the right inferior parietal lobule (IPL; MNI: 36, -42, 48; *t* = -5.55), the right supramarginal gyrus (SMG; MNI: 60, -24, 30; *t* = -5.54), and the right opercular part of the inferior frontal gyrus (IFGoperc; MNI: 54, 9, 18; *t* = -6.10) of the DAN; the left heschl gyrus (HES; MNI: -39, -21, 9; *t* = -5.30) and the right insula (INS; MNI: 39, -15, 9; *t* = -5.44) of the AN; the right lingual gyrus (LING; MNI: 18, -48, -6; *t* = *-*5.16) of the mVN; and the right precentral gyrus (PreCG; MNI: 24, -24, 63; *t* = -5.11) of the SMN (Table 2 and Fig. 2).

**Table 2:** Brain regions with decreased intranetwork FC in children with PWS compared to HCs (FWE-corrected *p* < 0.05 and a cluster size > 10 voxels)

Brain region	RSNs	Cluster size (voxels)	MNI coordinates of maximum voxel			Peak voxel *t*-value
			x	y	z	
Right IPL	DAN	23	36	-42	48	-5.5513
Right SMG	DAN	28	60	-24	30	-5.5385
Right IFGoperc	DAN	25	54	9	18	-6.1042
Right INS	AN	45	39	-15	9	-5.4412
Left HES	AN	29	-39	-21	9	-5.2980
Right LING	mVN	13	18	-48	-6	-5.1581
Right PreCG	SMN	24	24	-24	63	-5.1070

AN, auditory network; DAN, dorsal attention network; FC, functional connectivity; FWE, the familywise error; HCs, healthy controls; HES, heschil gyrus; IFGoperc, opercular part of inferior frontal gyrus; INS, insula; IPL, inferior parietal lobule; LING, lingual gyrus; MNI: Montreal Neurological Institute; mVN, medial visual network; PreCG, precentral gyrus; PWS, Prader-Willi syndrome; RSN, resting-state networks; SMG, supramarginal gyrus; SMN, sensorimotor network

### Altered internetwork functional connectivity

**Fig. 3:**
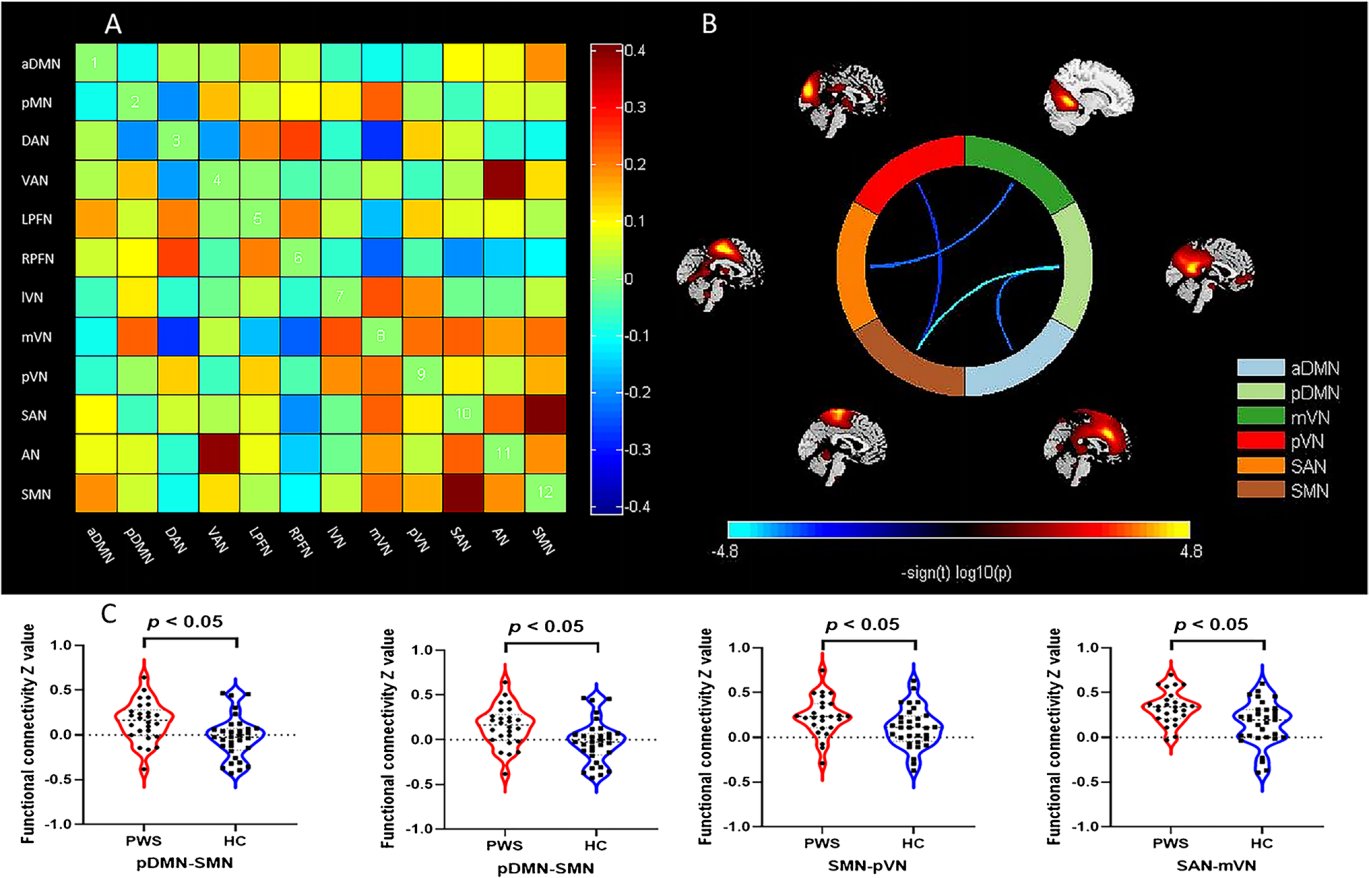
PWS group showed a decrease in internetwork functional connectivity compared to HCs by two-sample *t*-test (FDR-corrected *p* < 0.05). **A** Inter-network functional connectivity matrix;** B** and** C** Significant differences in intra-network functional connectivity between PWS compared with HCs. aDMN, anterior default mode network; AN, auditory network; DAN, dorsal attention network; FDR, the false discovery rate; HCs, healthy controls; lFPN, left frontoparietal network; lVN, lateral visual network; mVN, medial visual network; pDMN, posterior default mode network; pVN, posterior visual network; rFPN, right frontoparietal network; PWS, Prader-Willi syndrome; SAN, salience network; SMN, sensorimotor network; VAN, ventral attention network

In the PWS group, the four following pairs of networks showed decreased internetwork FC compared with those in HCs (Table 3 and Fig. 3): the pDMN and aDMN (*t* = -3.7647, *p* = 0.0004), pDMN and SMN (*t* = -4.7136, *p* = 0.0002), SMN and pVN (*t* = -3.5133, *p* = 0.0008), and SAN and mVN (*t* = -3.6999,* p* = 0.0005).

**Table 3:** Decreased internetwork FC in children with PWS compared to HCs (FDR-corrected* p* < 0.05)

FC	*p* value	*t* value
pDMN-aDMN	0.0004	-3.7647
pDMN-SMN	0.0002	-4.7136
SMN-pVN	0.0008	-3.5133
SAN-mVN	0.0005	-3.6999

aDMN, anterior default mode network; FC, functional connectivity; FDR, the false discovery rate; HCs, healthy controls; mVN, medial visual network; pDMN, posterior default mode network; pVN, posterior visual network; PWS, Prader-Willi syndrome; SAN, salience network; SMN, sensorimotor network

### Correlation analysis results

**Fig. 4:**
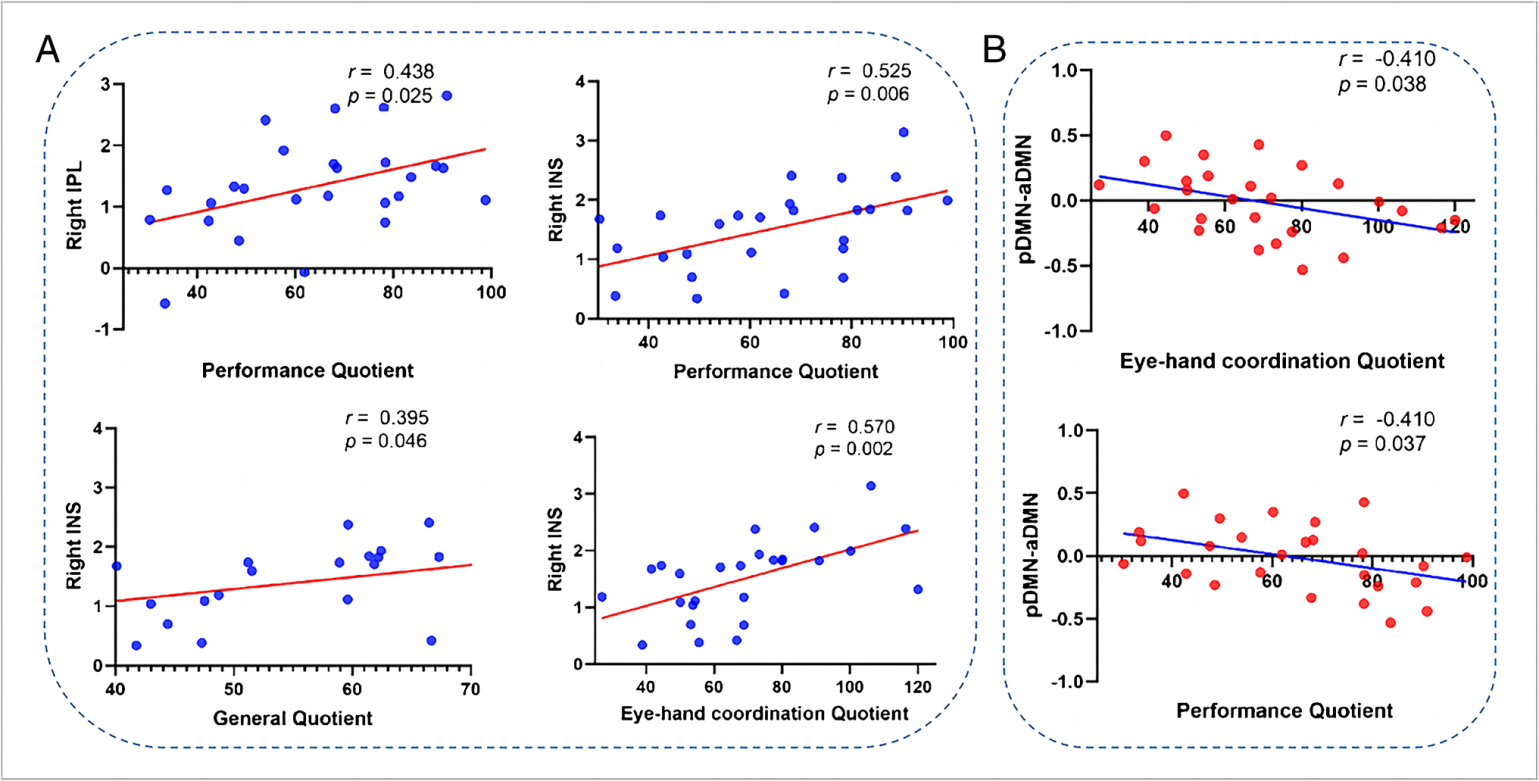
Significant correlations between intranetwork and internetwork functional connectivity and developmental variables in children with PWS. **A** Pearson correlation shows a increase between intra-network FC (right IPL of DAN and right INS of AN) and developmental quotients. **B** Pearson correlation shows a decrease between inter-network FC (pDMN-aDMN) and developmental quotients. aDMN, anterior default mode network; FC, functional connectivity; INS, insula; IPL, inferior parietal lobule; pDMN, posterior default mode network

We performed correlations between the intra/internetwork FC changes and developmental quotients (Fig. 4). For the intranetwork FC in the PWS group, the FC value of the right IPL of the DAN was positively correlated with the performance quotient (EQ, *r* = 0.438, *p* = 0.025), the FC value in the right INS of the AN was positively correlated with the EQ (*r* = 0.525, *p* = 0.006), the FC value in the right INS of the AN was positively correlated with the GQ (*r* = 0.395, *p* = 0.046), and the FC value in the right INS of the AN was positively correlated with the eye-hand coordination quotient (DQ, *r* = 0.570, *p* = 0.002). For the internetwork connectivity in the PWS group, the pDMN-aDMN FC value and DQ were negatively correlated (*r* = -0.410, *p* = 0.038), and the pDMN-aDMN FC value and EQ were negatively correlated (*r* = -0.410, *p* = 0.037).

### Intranetwork FC alterations as indicators

**Fig. 5:**
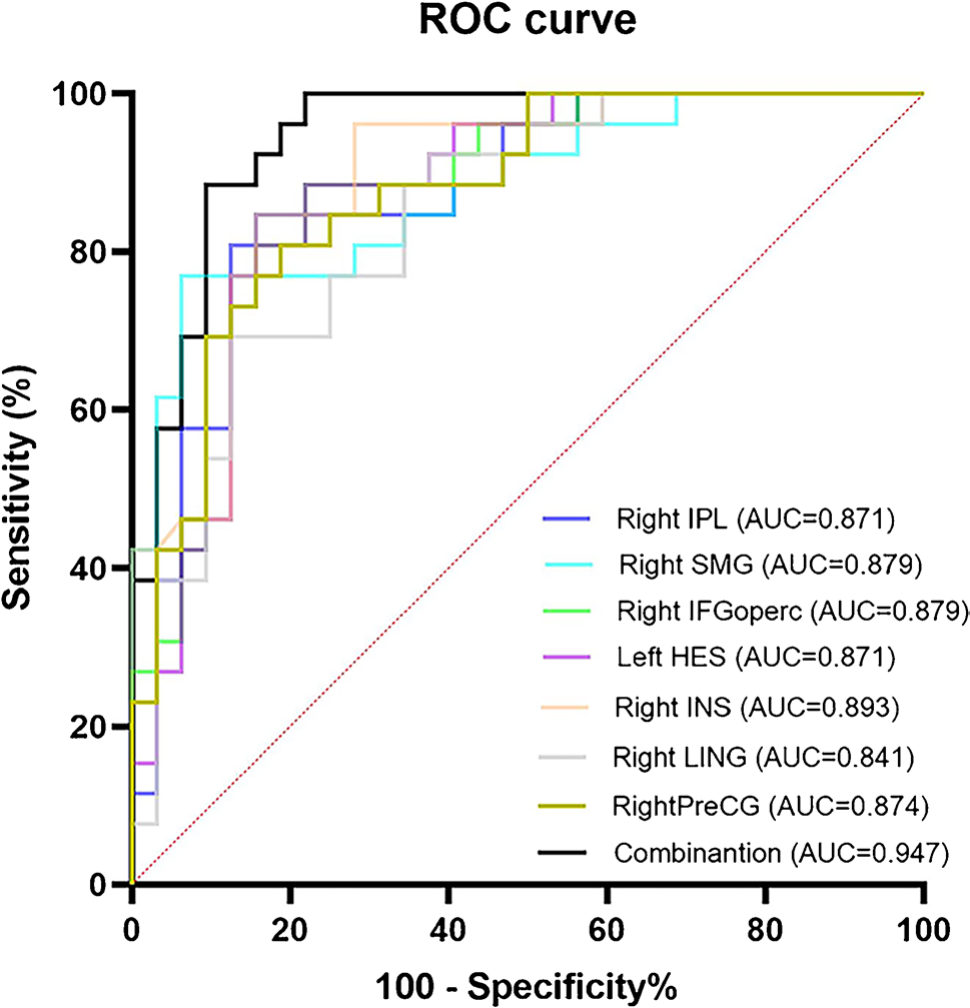
ROC curve for intranetwork FC alterations as indicators. AUC, area under the curve; CI, confidence interval; HES, heschil gyrus; IFGoperc, opercular part of inferior frontal gyrus; INS, insula; IPL, inferior parietal lobule; LING, lingual gyrus; PreCG, precentral gyrus; ROC, receiver operating characteristics; SE, standard error

Table 4 and Fig. 5 show the results of the ROC analysis. The combination of seven FC change regions of the four intranetworks in PWS led to an AUC of 0.947, a sensitivity of 96.15%, and a specificity of 81.25%. The resulting AUCs for the ROC curve values of the seven regions, namely, the right IPL, right SMG, right IFGoperc, left HES, right INS, right LING and right PreCG, were 0.871, 0.879, 0.879, 0.871, 0.893, 0.841 and 0.874, respectively.

**Table 4:** ROC curve analyses of the selected parameters

Variables	AUC	Sentivity (%)	Specificity (%)	SE	95% CI
Right IPL	0.871	80.77	87.50	0.0471	0.7791–0.9637
Right SMG	0.879	80.77	71.88	0.0458	0.7889–0.9683
Right IFGoperc	0.879	80.77	84.38	0.0449	0.7907–0.9665
Left HES	0.871	88.46	78.13	0.0470	0.7793–0.9635
Right INS	0.893	84.62	84.38	0.0417	0.8108–0.9741
Right LING	0.841	76.92	75.00	0.0512	0.7409–0.9418
Right PreCG	0.874	80.77	81.25	0.0452	0.7851–0.9625
Combination	0.947	96.15	81.25	0.0275	0.8932–1.000

AUC, area under the curve; CI, confidence interval; HES, heschil gyrus; IFGoperc, opercular part of inferior frontal gyrus; INS, insula; IPL, inferior parietal lobule; LING, lingual gyrus; PreCG, precentral gyrus; ROC, receiver operating characteristics; SE, standard error

## Discussion

### Altered FC within RSNs between the two groups

In the present study, the PWS group showed decreased FC in the DAN, AN, mVN and SMN compared to the HC group, and the FC of the right IPL of the DAN and the right INS of the AN in PWS were positively correlated with developmental scales.

Notably, the widespread disruption of the DAN and the stability of the VAN is similar to that observed in typical cognitive diseases such as Alzheimer's disease and type 2 diabetes [27–29], suggesting that these diseases may share a portion of attention deficit-related mechanisms. Clinically relevant cognitive decrements mainly occur mostly at two crucial stages in life: when the brain is developing in childhood, and when the brain suffers neurodegenerative changes associated with ageing. Our study concentrated on children with PWS and demonstrated functional impairments in their brain networks. In addition, structural brain abnormalities in adults with PWS reflect premature brain aging or abnormal brain development from another perspective [30]. Manning et al. [31] observed widespread increased cortical thickness in youth with PWS. The paradoxical results above may due to different time points and measurement methods of PWS samples. VN alterations in PWS have been widely reported in previous studies because studies of brain function in PWS have often used visual stimuli to investigate their effects on feeding behaviors [32, 33]. As an important part of the VN, the lingual gyrus primarily performs visual processing and unifies visual information to form conscious visual judgments [34]. A group of PWS patients showed hyperactivation in the visual brain areas when presented with a visual representation of disgusting food [32]. Decreased intranetwork FC in the VN was found in our results, contrary to the above results, which may be because our subjects were younger (before the emergence of binge eating) and brain regions associated with food stimulation were not overdeveloped at the time of the study. The SMN has also been an area of interest in PWS research. Pujol et al. [7] found increased FC within the primary sensorimotor cortex-putamen loop, which was closely associated with compulsive behaviors such as self-picking in PWS. Furthermore, based on the structural level, Ogura et al. [35] also found reduced volume in brain regions such as the precentral gyrus of the SMN, confirming that the tendency to compulsive symptoms is present at a very young age and is only apparent while growing up. Moreover, Yamada et al. [36] emphasized altered FC in specific brain regions, providing a clue to help decipher a constellation of ASD-like symptoms (compulsivity and insistence on sameness) in adults with PWS. Hence, these altered networks in PWS children may reflect underlying neurodevelopmental mechanisms of cognitive and behavioral abnormalities.

Beyond our expectations, our results found a largely symmetric decreased FC in the AN in children with PWS, which has rarely been reported before. The AN is generally considered to contain the primary auditory cortex (with the heschl gyrus as an important component), which transmits information to the secondary auditory cortex (with the superior temporal gyrus as an important component), which ultimately projects to higher perceptual processing brain areas via the thalamus. The temporal lobe is associated with the conversion of sensory input into derived meanings to maintain appropriate visual memory, language comprehension, and emotion association [37]. Meanwhile, the positive correlations between the intranetwork FC in the right insula of the AN and GDS quotients demonstrated that intranetwork FC changes in AN could be used as an indicator to screen for developmental delays in patients with PWS.

### Altered FC between RSNs between the two groups

In addition to the significant intranetwork FC changes observed in PWS, we also found significantly weaker FC between the pDMN and aDMN, pDMN and SMN, pVN and SMN, and mVN and SAN, and the correlation values between the pDMN and aDMN were negatively correlated with both DQ and EQ subdevelopmental scales.

The DMN was linked to cognitive and emotional control, being most active during rest and less active during task-based states involving attention or goal-directed behavior [38, 39]. The decreased FC value between the pDMN and aDMN is consistent with previous results [33] and is explained by the increased need for inhibitory control of PWS with the onset of binge eating. In addition, the pDMN and aDMN correlation values were negatively correlated with both DQ and EQ, laterally indicating that the higher the connection, the lower the PWS developmental scores and the more severe the symptoms in PWS. This phenomenon is completely opposite to the results of alterations in the intranetwork in the PWS network, which needs to be further investigated in conjunction with structural networks.

There is evidence that the FC within DMN, SMN and SAN does change significantly in individuals with PWS, possibly demonstrating small-scale intranetwork interactions [40]. Our results reveal internetwork interactions from macroscopic large-scale networks in children with PWS, speculateing that the FC reduction among these networks may be a reflection of the anomalous state of PWS. The correlation values of the pDMN and aDMN, which can reflect the development degree of PWS, and may be an important indicator for dynamic monitoring of PWS treatment effects in the future.

### Alterations in intranetwork FC values as indicators

ROC curve analysis revealed that core regions of DAN, AN, mVN and SMN had high sensitivity and specificity. Li et al. [27] showed that the activity of core regions in the DAN and DMN could be used as sensitive and specific biomarkers to distinguish Alzheimer’s disease using ICA and ROC curve analysis. Chen et al. [25] used ICA to find that the combined outcome of AN and DMN could predict the prognosis of tinnitus. Chodkowski et al. [41] identified that resting-state FC could identify neural models that were linked with obesity and eating behaviors during childhood. To date, there have been seldom studies of brain changes to identify individuals with PWS. Manning et al. [31] identified widespread alterations in neural structure of grey matter and cortex in young adults with PWS, suggesting possible developmental and maturational mechanisms. They then used machine learning of PWS brain structural changes to obtain brain-predicted age difference scores [30]. In our study, we found that combining these four RSNs resulted in high sensitivity and specificity for the disease with an AUC up to 0.947, indicating that this index can be used as a valuable imaging indicator for PWS screening and prognosis.

### Limitations

The present study has some limitations. First, considering for the unstable image signals of the cerebellar, a non-cerebellar template was used in our study. A prior study [42] emphasized that topographic patterns of volume differences in cerebellar structure contribute to altered FC in PWS, suggesting that the cerebellar network in PWS also palys an important role in PWS. Second, we used the correlational method to measure the temporal synchronization of the rs-fMRI time series, which does not provide information about the causal relationship between the RSNs in PWS. Other methods such as Granger causality, structural modeling technique and mutual information are needed to study the relationship between network alterations in PWS in the future. Although the sample size was sufficient to support the findings of this study, inherent bias were inevitable due to voluntary participation. Although the sample size was sufficient to support the findings of this study, inherent bias were inevitable due to voluntary participation. PWS subjects who were recruited may have more severe clinical manifestations and be more willing to seek treatment in the hospital, whereas those with milder conditions and a lower willingness to seek medical care were not enrolled.

## Conclusion

In conclusion, we identified decreased intranetwork and internetwork FC changes in children with PWS, with the main changes occurring in the DMN, DAN, AN, VN, SMN and SAN, these changes were associated with developmental scores. These findings reveal that reorganization of intrinsic brain FC occurs both within and between RSNs in children with PWS. Intranetwork FC changes in core regions might distinguish children with PWS from HCs. Thus, our findings provide new insights into the underlying neurodevelopmental mechanisms of PWS from a large-scale network perspective.
